# ChatGPT in Clinical Toxicology

**DOI:** 10.2196/46876

**Published:** 2023-03-08

**Authors:** Mary Sabry Abdel-Messih, Maged N Kamel Boulos

**Affiliations:** 1 Clinical Toxicology Centre Forensic Medicine and Clinical Toxicology Department, Faculty of Medicine Ain Shams University Cairo Egypt; 2 School of Medicine University of Lisbon Lisbon Portugal

**Keywords:** ChatGPT, clinical toxicology, organophosphates, artificial intelligence, AI, medical education

## Abstract

ChatGPT has recently been shown to pass the United States Medical Licensing Examination (USMLE). We tested ChatGPT (Feb 13, 2023 release) using a typical clinical toxicology case of acute organophosphate poisoning. ChatGPT fared well in answering all of our queries regarding it.

Since its public launch on November 30, 2022, ChatGPT, which ironically has not been specifically trained in medicine, has been taking the medical world by storm [1-3]. Developed by the San Francisco–based OpenAI Inc/LP, ChatGPT is a very large language model that uses deep learning artificial intelligence (AI) techniques to generate human-like responses to natural language queries. It is based on the Generative Pre-trained Transformer 3 (GPT-3 x) architecture, which has been trained on gigantic amounts of data. ChatGPT is currently being integrated into the Microsoft Bing search engine, which will soon make it readily accessible to hundreds of millions of online users worldwide, including patients, medical and nursing students, and clinicians [4].

We tested ChatGPT (Feb 13, 2023, release; standalone, available via OpenAI [5]) using a typical clinical toxicology vignette (a case of acute organophosphate poisoning) retrieved from an online presentation [6]. The case, as we modified it for and typed it in ChatGPT, as well as ChatGPT’s answer (which followed only a couple of seconds after inputting the case), are shown in Figure 1. The figure also shows a regenerated ChatGPT response after we pressed the “Regenerate response” button at the bottom of the first ChatGPT answer.

The clinical case example we used is a very straightforward one, unlikely to be missed by any practitioner in the field, and ChatGPT fared well in answering all of our queries regarding it. Both the first ChatGPT response and the regenerated one were fine and offered good explanations of the underlying reasoning. However, the pressing problem in real life is not one of finding the correct diagnosis but of taking appropriate history and being able to elicit and ascertain the correct signs. In real life, junior clinicians may arrive at the wrong diagnosis because they missed or confused the signs. As ChatGPT becomes further developed and specifically adapted for medicine, it could one day be useful in less common clinical cases (ie, cases that experts sometimes miss). Rather than AI replacing humans (clinicians), we see it as “clinicians using AI” replacing “clinicians who do not use AI” in the coming years.

**Figure 1:**
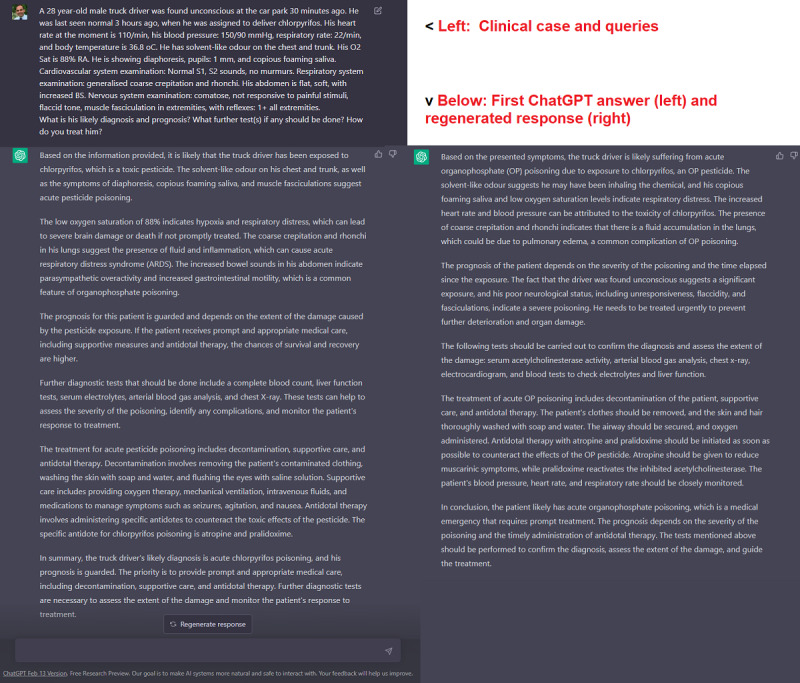
Diagnosing a case of acute organophosphate poisoning in ChatGPT.

## Abbreviations

AI: artificial intelligence
GPT-3 x: Generative Pre-trained Transformer 3
